# Amodal Presence and the Bounce/Stream Illusion

**DOI:** 10.1177/2041669518791833

**Published:** 2018-08-07

**Authors:** Stuart Anstis

**Affiliations:** Department of Psychology, UC San Diego, La Jolla, CA, USA

**Keywords:** amodal completion, attention, bounce/stream, motion

## Abstract

Ambiguous bounce/stream collision points were hidden behind an occluder so that observers had to complete them amodally. In Movie 1, straight or curved static lines were painted on the occluder. In Movie 2, dotted textures flowed in straight or curved lines across the front of the occluder. In Movie 3, moving eyes, painted on the occluder, either moved in straight lines, as if tracking streaming spots, or else followed curved paths, as if tracking bouncing spots. The straight (or curved) lines, texture flow or eye movements led to judgments of streaming (or bouncing). These effects demonstrate the role of attention and expectations in disambiguating bounce/stream stimuli.

Amodal completion occurs when several spatially separated elements in the visual input are perceptually combined into a single, partly hidden object (Burke, 1952; Davis & Driver, 2003; Michotte, 1991/2013). If a cat’s whiskers stick out on one side of a tree, and a cat’s tail on the other, we amodally complete a cat.

Most published studies have examined amodal completion of stationary objects. But here, we study the interaction between amodal completion and the stream/bounce illusion (Grove, Robertson, & Harris, 2016). This illusion is an ambiguous bistable motion display in which two disks approach each other on a collision course. The display can be perceived as two disks either streaming through each other or bouncing off each other. We added a stationary occluder that covered the collision point, such that the colliding motion was amodally completed behind the occluder. We found that painting stationary lines on the occluder, or adding dot texture or stylized eyeballs that swiveled to follow the presumed hidden motion, could strongly affect the probability of seeing streaming or bouncing motion.

Movie 1 shows two identical pairs of moving disks, exposed in alternation on left and right. The occluder in the center of the left-hand display had a stationary letter X painted on it, and as a result, the two black disks appeared to stream past each other as they disappeared behind the occluder and re-appeared. The occluder in the right-hand display had two back-to-back letter C’s painted on it. As a result, these perceptually diverted the paths of the disks and made them appear to bounce off one another.

**Movie 1 (Click to play).** The disks move in the same way behind each occluder. But the X on the left-hand occluder induces the appearance of *streaming* movement, while the curved lines on the right-hand occluder induce the appearance of *bouncing* movement.

This is reminiscent of Shepard and Zane’s (1983) path-guided motion, in which a curved gray path, briefly flashed between two alternately displayed black dots, induced the illusion of a single dot moving back and forth over that path. But Shepard’s curved line was a briefly flashed integral ingredient within an apparent-motion sequence. The lines on our occluders were stationary and visible at all times and did not partake directly in the motion but merely steered it, presumably via the observer’s attention.

Movie 2 presents moving dotted textures whose motion trajectories are similar to the static lines in Movie 1. On the left in Movie 2, the dots move along straight lines and promote streaming, while on the right the dots move along curved paths and promote bouncing.

**Movie 2 (Click to play).** The disks move in the same way behind each occluder. But the dotted textures on the left-hand occluder sweep obliquely and induce the appearance of *streaming* movement, while the textures on the right-hand occluder follow curved paths and induce the appearance of *bouncing* movement.

Movie 3 shows two identical pairs of moving disks, but now each occluder bore two moving eyeballs. The upper eyes swiveled to track the left-hand disk, and the lower eyes tracked the right hand disk. In the left-hand display, the upper eye swept obliquely down from top left to bottom right, while the lower eye swept obliquely down from top right to bottom left. These are the eye movements that observers would make if they were following streaming disks. As a result, the disks appeared to stream past each other.

**Movie 3 (Click to play).** The disks move in the same way behind each occluder. But the stylized eyes on the left-hand occluder sweep obliquely and induce the appearance of *streaming* movement, while the eyes on the right-hand occluder follow curved paths and induce the appearance of *bouncing* movement.

In the right-hand display, the eyes followed curved paths. The upper eye swept down from top left to the center, then changed direction and swung down to the bottom left. Correspondingly, the lower eye swept down from top right to the center, then veered down to the bottom right. These are the eye movements that observers would make if they were following bouncing disks. As a result, the disks appeared to bounce off each other. There has been much debate about the role of eye movements in guiding social attention (e.g., Freeth, Foulsham, & Kingstone, 2013). We are not claiming any special status here for human eyes in driving bounce/stream movements.

Amodal completion usually joins spatially separated, stationary fragments into a single partly hidden surface. We show here that amodal completion can also organize spatially separate moving disks that arrive at different times. It can select different perceptual motion paths from an ambiguous motion input, and this selection process is largely under the control of top-down attention and expectations. The markings on the occluders have no physical or logical connection to the moving disks, but they do have a perceptual connection.
